# Oxidative stress-induced formation of covalently linked ribulose-1,5-bisphosphate carboxylase/oxygenase large subunit dimer in tobacco plants

**DOI:** 10.1186/s13104-019-4153-z

**Published:** 2019-02-28

**Authors:** Jasmina Kurepa, Jan A. Smalle

**Affiliations:** Plant Physiology, Biochemistry, Molecular Biology Program, Department of Plant and Soil Sciences, College of Agriculture, Food and Environment, University of Kentucky, Lexington, KY 40546 USA

**Keywords:** Rubisco, Oxidative stress, Dimer, Paraquat, Titanium dioxide nanoparticles

## Abstract

**Objective:**

Many abiotic stresses cause the excessive accumulation of reactive oxygen species known as oxidative stress. While analyzing the effects of oxidative stress on tobacco, we noticed the increased accumulation of a specific protein in extracts from plants treated with the oxidative-stress inducing herbicide paraquat which promotes the generation of reactive oxygen species primarily in chloroplasts. The primary objectives of this study were to identify this protein and to determine if its accumulation is indeed a result of oxidative stress.

**Results:**

Here we show that the paraquat-induced protein is a covalently linked dimer of the large subunit of ribulose-1,5-bisphosphate carboxylase (LSU). Increased accumulation of this LSU dimer was also observed in tobacco plants exposed to ultra-small anatase titanium dioxide nanoparticles (nTiO_2_), which because of their surface reactivity cause oxidative stress by promoting the generation of superoxide anion. nTiO_2_ nanoparticle treatments also caused a decline in the chloroplast thylakoid proteins cytochrome f and chlorophyll a/b binding protein, thus confirming that covalent LSU dimer formation coincides with loss of chloroplast function.

**Electronic supplementary material:**

The online version of this article (10.1186/s13104-019-4153-z) contains supplementary material, which is available to authorized users.

## Introduction

Ribulose-1,5-bisphosphate carboxylase/oxygenase (Rubisco) catalyzes the rate-limiting step of CO_2_ fixation in photosynthesis and is thus the key enzyme in the global carbon cycle [1]. It is a multi-subunit enzyme complex composed of eight small and eight large subunits (LSU) with the latter assembling in the complex as LSU dimers that contain the active sites [2]. Rubisco, being a catalytically very inefficient enzyme, became an important target for improving photosynthetic efficiency, an effort that involved intense research aimed at understanding the regulation of Rubisco catalytic mechanisms, holoenzyme assembly mechanisms and pathways that lead to complex disassembly and degradation [3–6].

Degradation of Rubisco is an important catabolic process. Because of its abundance, Rubisco is the most significant cellular storage of nitrogen and the remobilization of this nitrogen by proteolysis of Rubisco is a hallmark of senescence and stress responses [7]. The current view of Rubisco degradation indicates that the choice of proteolytic pathway may largely be dictated by which endogenous and environmental factors are triggering the stress response [8]. In addition to fragmentation, it was shown in the aquatic monocot *Lemna gibba* that small and large Rubisco subunit can form covalently linked dimers after exposure to ultraviolet radiation stress [9].

Here we describe the formation of covalently linked LSU dimers in burley tobacco exposed to oxidative stress. Oxidative stress was induced by the reactive oxygen species (ROS)-generating herbicide paraquat (PQ) and by titanium dioxide nanoparticles (nTiO_2_). PQ is a redox-active herbicide that accepts electrons from photosystem I and transfers them to oxygen thus loading the cell with superoxide radicals [10]. Superoxide radicals are further metabolized into H_2_O_2_ and in turn, into hydroxyl radicals. All these ROS damage cellular components and induce the oxidative stress response [11–13]. ROS-induced damage is also an important component of nanomaterial-induced toxicity. Due to the increased use of nanomaterials in all areas of technology and thus their presence in the biosphere, analyses of their mechanisms of nanotoxicity have been intensified and in recent years, they showed that one of the common mechanisms of toxicity is the generation of ROS and concomitant oxidative stress [14–16]. Exposure to nTiO_2_ for example leads to increased intracellular ROS in cells of all tested species and leads to cellular damage in function of the size, dose and surface reactivity of the nanoparticles used [17–20].

## Main text

### Materials and methods

#### Plant growth

Burley tobacco seeds (KT 204LC variety) were obtained from F.W. Rickard Seeds, Inc. (Winchester, KY). Seeds were sterilized (5 min in 70% ethanol, followed by 20 min in 50% commercial bleach solution and 3 rinses in sterile water) and sown on Murashige and Skoog medium with 3% sucrose (pH 5.7). Plants were grown in axenic cultures in a controlled environment chamber in continuous light (25 °C; 80 µmol m^−2^ s^−1^).

#### Treatments

Plants were analyzed when 2 months old. For all experiments, the laminar part of mature leaves of three plants were pooled per sample and each treatment was done in triplicate. Paraquat (Sigma, methyl viologen) stock was prepared as a 100 mM aqueous solution. Anatase nTiO_2_ (5–15 nm, 15 wt% nanopowder dispersion; US Research Nanomaterials) stock was prepared by diluting the commercial suspension in methanol (1:9 v/v). Immediately before treatments, the stock was further diluted in distilled water to a final concentration of 2 mM and sonicated for 5 min.

#### Immunoblotting analyses

Protein extraction and immunoblotting analyses were performed as previously described [21]. The primary antibodies used were: anti-Rubisco LSU antibody (RbL form I and II, Agrisera, dilution 1:10,000), anti-Chlorophyll a/b binding protein (Cab) antibody (Lhcb1, Agrisera, 1:10,000), anti-Cytochrome f (Cyt f) antibody (PetA, Agrisera, 1:10,000), anti-Heat Shock Protein 90 (HSP90) antibody (at-115, Santa Cruz Biotechnology, 1:5000) and anti-Binding Immunoglobulin Protein (BiP) antibody (at-95; Santa Cruz Biotechnology, 1:5000). The secondary antibody used was horseradish peroxidase-conjugated anti-rabbit IgG goat antibodies obtained from Santa Cruz Biotechnology. Immunoblots were developed using SuperSignal West Femto substrate (Thermo-Pierce) using a ChemiDoc™ XRS molecular imager (Bio-Rad).

#### Protein identification

SDS-PAGE gels for protein identification by mass spectrometry were prepared following the published guidelines [22]. After separation, proteins were stained with Coomassie Brilliant Blue R-250, destained and the band of interest was excised from the gel. After extensive washing with water, the sample was submitted for analysis. Mass spectrometric analysis was performed at the Proteomics Core Facility of the University of Kentucky. The protein was digested with trypsin and peptides were extracted and analyzed by LC/MS/MS on an Orbitrap mass spectrometer. The resulting spectra were submitted for a database similarity analysis and the matches were ranked by score. The search program, MASCOT, was adjusted to analyze the “other green plants” database.

### Results

**Fig. 1:**
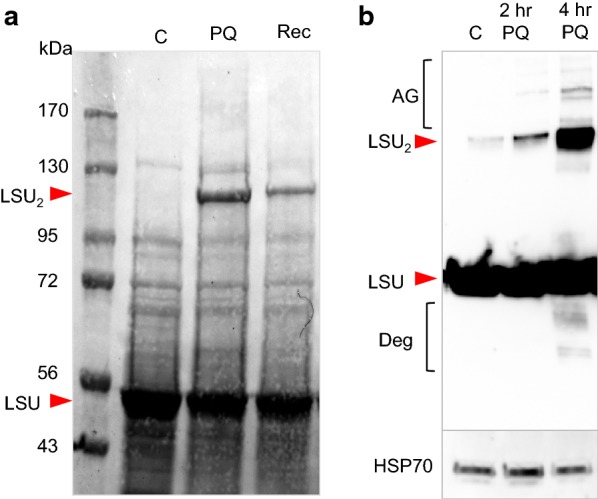
Formation of LSU dimer after paraquat (PQ) treatment. **a** Plants were incubated for 4 h in water (control, C) or 100 µM PQ (PQ and Rec). The recovery (Rec) sample was incubated in water for an additional 4 h. Protein extracts were resolved on a 7.5% separating acrylamide gels and transferred to nitrocellulose membrane. The Ponceau S staining of the membrane is shown. LSU, the large subunit of Rubisco. The size of the protein mass markers is shown on the left. **b** Plants were treated for 2 and 4 h with 100 µM PQ. Total protein extracts were resolved on a 7.5% separating acrylamide gels and transferred to nitrocellulose membranes. The membranes were probed with anti-LSU antibodies or anti-HSP70 antibodies (loading control)

Young leaves of 2-month-old sterile grown burley tobacco plants were incubated in water or 100 µM aqueous solution of PQ in light for 4 h. After that, one PQ-treated sample was washed and incubated in water for another 4 h to test for recovery. Analyses of total protein extracts resolved on SDS/PAGE gels showed that PQ treatment leads to a marked induction of a ~125 kDa protein that can be observed after total protein extracts transferred to nitrocellulose membranes were stained by Ponceau S, which is a reversible protein dye with low sensitivity (Fig. 1a). To identify the PQ-induced protein, we ran preparative denaturing gels, excised the induced protein and identified it using mass spectrometry. These analyses revealed with high certainty (64% coverage) that the accumulated protein is Rubisco LSU. Using the MASCOT search program, the top hit was LSU from *Nicotiana debneyi* (Additional file 1). There were 469 other significant Rubisco matches (as a testimony to sequence conservation of this protein among plant species). On denaturing SDS-PAGE protein gels, LSU has an apparent molecular mass of 55 kDa (Fig. 1a). Because the oxidative stress-induced LSU protein was approximately twice the size of the LSU monomer, we concluded that this LSU version is a covalent dimer.

Next, we performed immunoblotting analyses to confirm that the induced protein is indeed LSU. We treated leaves of 1-month-old in vitro grown tobacco plants with 100 µM PQ for 2 or 4 h (Fig. 1b). Immunoblotting analyses of total protein extracts showed that increased treatment time led to an increase in LSU dimer level. In addition, immunoblotting analyses showed that both high molecular weight aggregates as well as degradation products of LSU are formed during persistent chloroplast-generated oxidative stress (Fig. 1b).

**Fig. 2:**
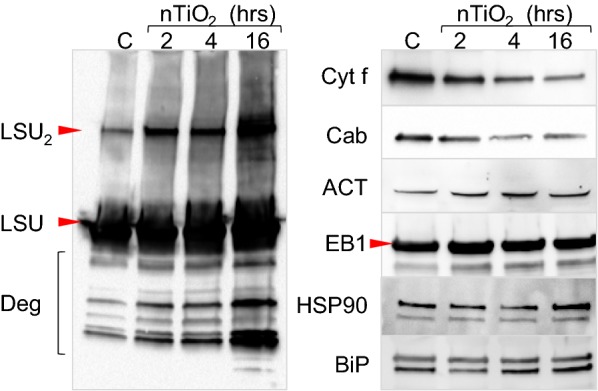
Formation of LSU dimer after nanoparticle treatment. Plants treated for 2, 4 or 16 h with ultra-small anatase TiO_2_ nanoparticles (nTiO_2_) were used for the isolation of total protein extracts. Protein extracts were resolved on 4–20% gradient separating acrylamide gel and transferred to a nitrocellulose membrane. The membranes were probed with antibodies specific for the protein(s) indicated on the left-hand side of each immunoblot. Deg, degradation products

Next, we tested whether another oxidative stress inducer can also lead to the formation of a covalently linked LSU dimer and chose to test the effect of exposure to nTiO_2_. For our experiments, we used a sublethal dose of ultra-small (aggressive and cell wall- and membrane-permeable) anatase nTiO_2_ [20, 23]. We incubated excised leaves of 2-month-old in vitro grown tobacco plants in water or an aqueous suspension of 2 mM nanoparticles for different times. Immunoblotting analyses revealed that the denaturation-resistant LSU dimer is indeed formed and accumulates to a high level after prolonged (>12 h) treatments with nTiO_2_ (Fig. 2). Total protein extracts of nTiO_2_-treated tobacco were also analyzed using antibodies against the chloroplast thylakoid proteins chlorophyll a/b binding protein (Cab) and cytochrome f (Cyt f). Both proteins were less abundant in extracts of the nanoparticle-treated leaves, suggesting that nanoparticles impacted the overall function of tobacco chloroplasts (Fig. 2). In contrast, the levels of cytoskeletal proteins actin and EB1 and the levels of chaperoning proteins HSP90 and BiP did not change in response to the nanoparticle treatments (Fig. 2).

### Discussion

Burley is a mutant tobacco variety with characteristic yellow–green leaves. Although the genetic nature of this leaf phenotype remains unknown, it has been recently shown that key genes related to chlorophyll biosynthesis and photosynthesis are significantly down-regulated in burley plants and that this causes a decrease in chlorophyll content and a reduction of photosynthesis efficiency when compared to other tobacco varieties [24]. In previous studies, we have analyzed total protein extracts of other tobacco varieties (e.g., Petit Havana SR1) and of the model plant Arabidopsis exposed to oxidative stress and we did not observe any accumulation of covalently linked LSU dimer in total extracts [25–32]. However, the presence of a stress-induced covalently linked LSU dimer was inadvertently shown in a study describing the effects of UV treatments on Rubisco stability in *L. gibba* [9]. This study describes the stress-induced formation of covalent dimers of the large and small Rubisco subunits in duckweed and a number of terrestrial monocots and dicots [9]. It is now well-established that ultraviolet irradiation causes oxidative stress [33]. Thus, one can hypothesize that the formation of covalently linked LSU dimer is an aspect of oxidative stress-induced damage in plant chloroplasts and that due to the nature of the genetic make-up of burley, this phenomenon can be more easily observed in this tobacco variety.

### Limitations

In addition to the question if oxidative stress-induced covalent LSU dimer linkage is universal to all plants, we have unanswered questions about the mechanism of the stress-induced covalent LSU dimer formation. We currently do not know if oxidative stress promotes the formation of the covalent bonds between LSU subunits between specific amino acid residues or for example, between a less defined number of oxidized amino acids.

## Additional file

**Additional file 1.** Database similarity analysis. The PQ-induced protein (Fig. 1a) was trypsin-digested and the peptides were analyzed by LC/MS/MS on an Orbitrap mass spectrometer. The resulting spectra were submitted for a database similarity analysis, and the matches were ranked by score.

## Abbreviations

BiP: Binding Immunoglobulin Protein
Cab: chlorophyll a/b binding protein
Cyt f: cytochrome f
HSP90: Heat Shock Protein 90
LSU: large subunit of ribulose-1,5-bisphosphate carboxylase/oxygenase
nTiO_2_: titanium dioxide nanoparticles
PQ: paraquat
Rubisco: ribulose-1,5-bisphosphate carboxylase/oxygenase
